# Illuminating innovations: leveraging the optoelectronic capabilities of carbon dots for advanced displays, sensors, and renewable energy solutions

**DOI:** 10.3389/fchem.2026.1843453

**Published:** 2026-08-04

**Authors:** Azkah Shehzadi, Tanzeela Fazal, Mazloom Shah, Saima Daud, Sajid Mahmood, Salah Knani, Reem Alreshidi, Shahid Iqbal

**Affiliations:** 1 Department of Chemistry, Abbottabad University of Science and Technology, Abbottabad, Khyber Pakhtunkhwa, Pakistan; 2 Department of Chemistry, Faculty of Science, Grand Asian University Sialkot, Sialkot, Pakistan; 3 Low Dimensional Materials Research Center at Khazar University, Baku, Azerbaijan; 4 Center for Scientific Research and Entrepreneurship, Northern Border University, Arar, Saudi Arabia; 5 Department of Physics, College of Science, Northern Border University, Arar, Saudi Arabia; 6 Department of Chemical and Environmental Engineering, University of Nottingham Ningbo China, Ningbo, China

**Keywords:** carbon dots, graphene quantum dots, light emitting diodes, optoelectronic, photostability

## Abstract

Carbon dots (CDs) are a fascinating area of research. Carbon nanodots (CDs), also known as carbon quantum dots (CQDs), carbon nanodots (CNDs), and graphene quantum dots (GQDs), have garnered increasing attention in recent times due to their accessibility, non-toxicity, and affordability as carbon-based materials. This paper highlights several synthetic approaches for creating CDs, including hydrothermal synthesis, pulsed laser ablation, direct carbonization, microwave and ultrasonic aided chemical synthesis, and electrochemical methods. Moreover, CDs also possess excellent photostability as they can maintain their optical properties like photoluminescence (PL), charge transfer properties, etc., even under prolonged light exposure. Owing to their distinct optical and optoelectronic characteristics, C-dots have the potential to serve as crucial components for several fields, including optoelectronics, solar cells, light-emitting diodes, and sensitizers. In addition to encouraging the practical implementation of CDs in many current and future research lines, we hope that the material presented in this review will stimulate further fascinating study on CDs from a fresh angle.

## 1 Introduction

Among the elements that are most prevalent in the crust of the planet is carbon. Numerous useful materials, including different types of nanomaterials, are based on carbon (Paper and Paper, 2002). Since nanotechnology has advanced so much in recent years, carbon-based materials have transformed from cheap waste to valuable commodities. Adding value is often accomplished via the use of novel synthesis techniques, which enable the simple and controlled manufacture of nanomaterials (Kwee et al., 2021; Lu et al., 2012; Marinovic et al., 2017). The sophisticated subclass of carbon-based nanomaterials known as carbon dots (CDs) was first created in 2004 (Li et al., 2012; Xu et al., 2004). Since carbon nanotubes (CNDs) are believed to consist of sp2/sp3 carbon and oxygen/nitrogen-based groups or polymer groups, CNDs are believed to have more practical uses in a range of fields, such as photocatalysis, photothermal therapy, electrochemical energy storage, biological imaging, light-emitting devices, and pH sensing for accuracy in the biomedical and visual fields (Li et al., 2021). To date, several types of CDs, such as CNDs, GQDs, polymer dots (PDs), and so on have been synthesized (Ghosh et al., 2021; Lee et al., 2021; Lin et al., 2020; Sakthivel et al., 2021; Verma et al., 2021).

**FIGURE 1:**
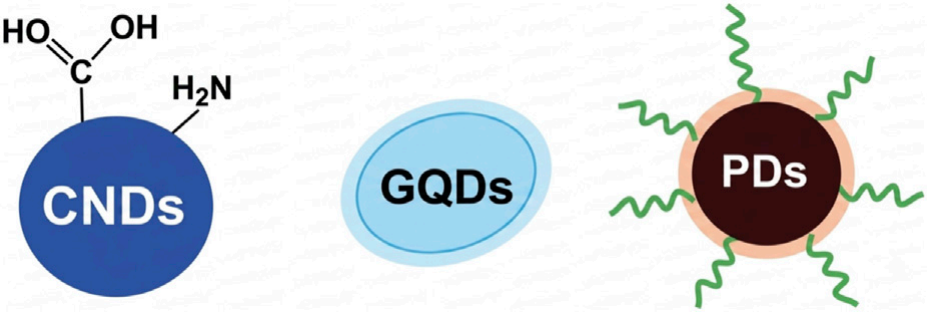
Different types of CDs (Behi et al., 2022).

A “water-in-oil” emulsion was employed by Rhee and Kwon as a nanoscale self-assembling platform in their initial size-controlling synthesis procedure for CNDs (Kwon et al., 2014; Wang X. et al., 2010). The resulting water micelles were encased in an immiscible oil by surfactant molecules. The temperature was then raised to 250 ^o^C for 2 hours while under an argon atmosphere to generate the CNDs. To optimize the creation of CNDs, they also investigated the effects of two distinct temperatures, 160 ^o^C and 200 ^o^C. In addition, the proper temperature was maintained throughout the CND carbonization and capping operations. The concentration of water-surfactant may control the micelle size, it was discovered. Therefore, this approach may provide size tenability with a limited size dispersion. Furthermore, because of their non-identical band gaps, Rhee and Kwon found that different-sized CNDs had different PL behaviors. The blue shift of the PL peak was about 25 nm as the CNDs grew from 1.5 to 3.5 nm. The sp^2^ hybridization exhibiting different energy gaps due to its association with oleylamine ligands, which acted as auxo chromes to reduce the energy gaps, provided another explanation for the differing results of the quantum size effect (Figure 1). Band gaps were thus larger in large CNDs than in small CNDs with relatively modest ligand/sp^2^ clusters. This resulted in the large absorption peak at 370 nm being obscured by enormous CNDs and the highest PL peak being shifted to 360 nm (Behi et al., 2022).

The sensor community has focused a lot of interest on CNDs because of their outstanding luminous performance, low cost, low toxicity (Singh et al., 2020), strong biocompatibility (Kim and Kim, 2020), optoelectronics (Astafiev et al., 2021), energy (Bian et al., 2019), chemical stability, photocatalysis (Wang et al., 2020) and biological imaging (Gao et al., 2017). Unmodified CNDs often handle a single physics-chemical property due to the absence of particular groups, which surely restricts their wider uses, including full-color displays, light-emitting diodes (LEDs), and bioimaging (Yang Y. et al., 2021; Wang et al., 2018). Thus, creating CNDs with multicolored emissions is desired (Zhang et al., 2023). The following describes how CDs vary from other carbon nanostructures. Due to its favorable environmental effects, ease of synthesis or fabrication, and widespread commercial availability, carbon compounds such as graphite, graphene, carbon nanotubes, fullerene and diamond have historically found extensive application in the field of material science. The lack of an appropriate band gap makes the macro-sized carbon material challenging to use as an ideal luminous material (Tao et al., 2019). By reducing the size up to dot level, bandgaps can be easily tuned over a wide range. CNDs represent a highly appealing substitute carbon source for the “older generation” of well-researched carbon nanostructures. The polar groups that are formed from the starting materials during their production allow these flexible nanocarbon compounds to also be well-dispersed in water (Goryacheva et al., 2017).

Due to the appealing feature of having electrochemical characteristics that are equal to graphene, GQDs are used far more often than other nanoparticles in a variety of fields, including photocatalysis, biosensing, optoelectronic instruments, bioimaging applications, and biological and environmental purposes (Hassanvand et al., 2021; Karimzadeh et al., 2018). CNDs are characterized primarily by the quantum confinement effect (QCE), which manifests when the CNDs’ size is less than the Bohr exciton radius. The valence band and conduction band transition are covered in the QCE phenomenon. To put it briefly, continuous energy bands become discrete energy levels as a material’s size drops to the nanoscale and the bandgap rises as a result. When the CNDs have insufficient surface chemical groups and a large, conjugated p-system, the QCE of the conjugated p-electrons is what causes fluorescence. The carbon core states are believed to have a fluorescence center located in the bandgap of the conjugated p-system. Moving toward the red region is the emission peak due to the larger conjugated p-system size. The conjugated p-system with limited size affects how the valence band and conduction band of CNDs split apart (Kwon et al., 2014; Sk et al., 2014). Subsequently, the electrons go straight to the valence band’s empty state. It is known as the fluorescence bandgap and results in the electron and hole recombining simply. It is projected that the hue of the CNDs emissions and their variable bandgap will alter with modifications to the size of the conjugated a-systems. Reaction conditions might be changed to increase the conjugated p-system size (Sekar et al., 2021).

This review’s main goal is to present a thorough and critical evaluation of the main carbon dot (CD) synthesis techniques, such as hydrothermal, microwave-assisted, laser ablation, electrochemical, and direct carbonization methods. This review assesses how each synthesis route affects the structural, optical, and optoelectronic properties of CDs, including particle size, photoluminescence behavior, quantum yield, surface functionalization, and charge-transfer characteristics, rather than just listing the various methods. The performance of CDs in optoelectronic applications, including as light-emitting diodes (LEDs), solar cells, sensors, and related devices, is also strongly correlated with these synthesis-dependent features. In order to offer recommendations for choosing suitable fabrication techniques and to determine future research paths, a comparative study highlighting the benefits, drawbacks, and applicability of each synthesis approach is offered.

### 1.1 Meta-analysis of publication trends

**FIGURE 2:**
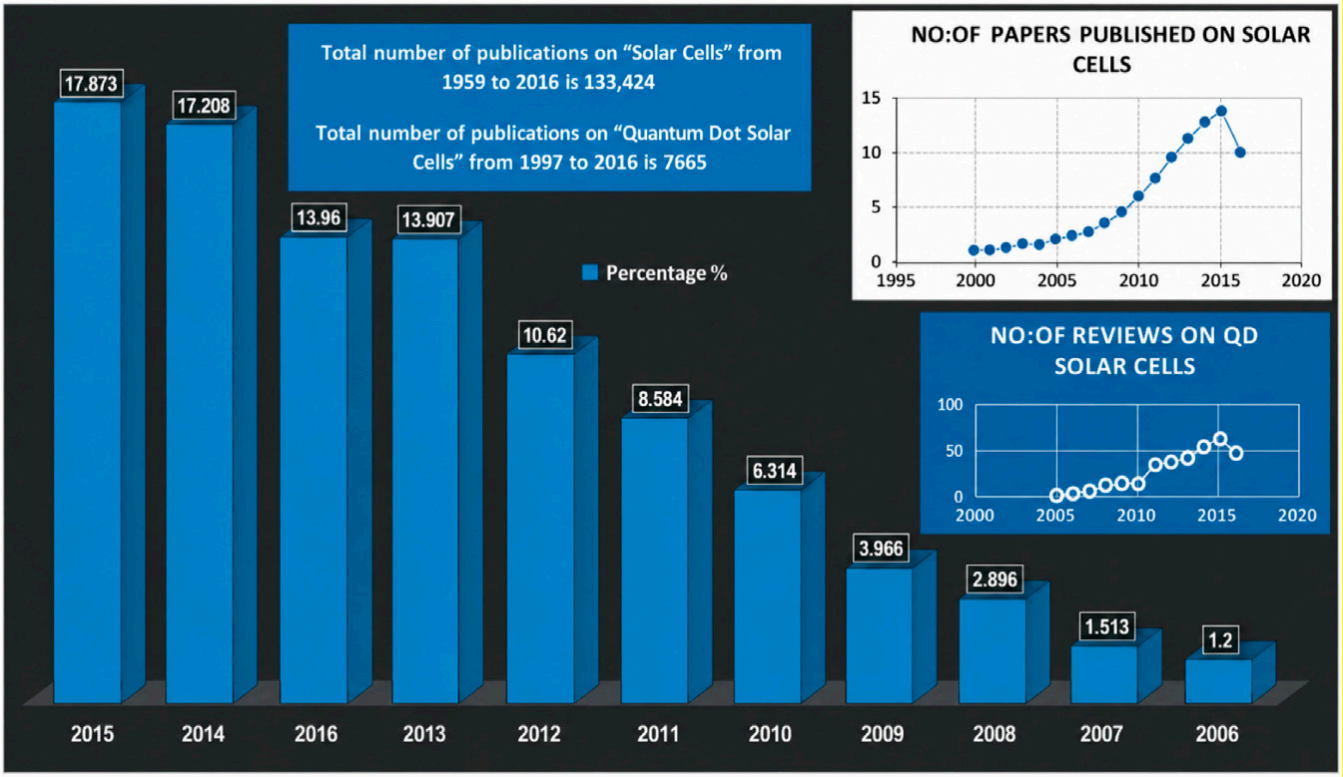
Over a 10-year period, a systematic distribution curve displaying the total yearly number of publications containing the indexed keywords “Carbon Dots/Graphene Quantum Dots” paired with “Optoelectronics/LEDs/Solar Cells” was created.

The global amount of scholarly literature on the optoelectronic uses of Carbon Dots (CDs) during the last 10 years is shown in Figure 2 to demonstrate the scientific advancement in this topic.

## 2 Synthesis of carbon nanodots (CNDs)

The first time CNDs were reported by Xu et al., in 2004, as a result of unintended discovery (Xu et al., 2004). Carbon nanotubes were reported for the experiment by using arc-discharge soot as a source. During the procedure, the components of the soot solution were separated using gel electrophoresis, which made a new band of fluorescent material visible. CNDs are now synthesized by a variety of physical and chemical procedures (Sakthivel et al., 2021). A fresh band of fluorescent material became evident throughout the process when the soot solution’s components were separated using gel electrophoresis. These days, CNDs are produced using a range of physical and chemical techniques (Khan et al., 2021; Xu et al., 2015). Two main types of CND synthesis methodologies are i) top-down and ii) bottom-up approaches. Top-down methods concentrate on utilizing chemical, electrochemical and other methods to break larger carbon structures down into nanoscale structures (Goryacheva et al., 2017; Hassanvand et al., 2021; Karimzadeh et al., 2018) or physical techniques such as arc discharge (Sk et al., 2014), laser ablation (a straightforward method for creating CQDs are laser-based) (Behi et al., 2022; Sekar et al., 2021), etc. However, bottom-up methods include creating CNDs from smaller carbon units (small organic compounds) primarily by chemical synthesis, which includes the template technique (Khan et al., 2021; Nguyen et al., 2015; Xu et al., 2015), and hydrothermal and microwave synthesis (De Medeiros et al., 2019; Mitra et al., 2012; Roy et al., 2015; Shi et al., 2016).

Top-down approaches, in general, make it possible to produce CNDs with better features and consequently, performance. But they also have some drawbacks, such as a lower fluorescence quantum yield, challenging collection, complex processes, and expensive instruments. Bottom-up approaches offer the advantage of simpler controllable synthetic conditions, a greater availability of carbon sources that are suited for synthesis and generally better fluorescence quantum yields in the generated CNDs. This approach’s sole drawback is that it requires more time and effort for the subsequent purification stages, which has a major impact on CND performance. These days, the most popular synthesis strategy is the bottom-up approach.

### 2.1 Top-down synthesis

For top-down approaches (Table 1), the most widely used techniques for producing carbon-based nanomaterials are probably laser ablation and arc discharge. An arc discharge occurs when two electrodes, often graphite rods, are linked to a current that vaporizes the electrodes. This results in the production of soot, which may include various carbon-based nanoparticles. By exposing a solid surface to radiation, a process known as pulse laser ablation releases nanomaterials. A quick and inexpensive method for synthesizing nanomaterials, laser ablation in solution (LAS) single-step top-down technique has drawn attention. Low contamination and byproduct formation rates are only two of LAS’s benefits (Nguyen et al., 2015). Gonçalves et al. produced passivated CNDs by laser ablation in a solution containing NH2-polyethylene glycol (PEG200), N-acetyl-l-cysteine (NAC), and water. Nguyen et al. have evaluated this strategy’s many facets and how they affect CNDs. The mean size of CNDs, for example, decreases with decreasing laser fluence and spot size. Moreover, extending the time of the irradiation reduces CNDs and produces several functional groups (Nguyen et al., 2015). For arc discharge, laser ablation and high-energy ion beam radiation, precursors including graphite powder and carbon-based cement have been frequently employed (Li et al., 2012).

**TABLE 1:** There are a few top-down and bottom-up synthetic techniques for CDs with very good control on synthesis are discussed (Khan et al., 2021).

Sources	Synthetic approaches	Size (nm)	Applications	Ref
Glucosamine hydrochloride	Microwave synthesizer	2.0 ± 0.7 nm	ROS scavenging	Ji et al. (2019)
Citric acid and ethylene diamine	Hydrothermal	1.9 ± 0.2 nm	Theranostic cancer	Wu et al. (2020)
Citric acid and polyethyleneimine	Hydrothermal	9.8–9.9 nm	Bioimaging *in vivo* and photothermal	Sun et al. (2019)
Citric acid and polyethyleneimine	Microwave	-5 nm	Enhancement of bioavailability of curcumin	Arvapalli et al. (2020a)
Milk	Hydrothermal	−20 nm for CNDs doxorubicin complexes	Drug delivery	Yuan et al. (2017)
Exopolysaccharides	Hydrothermal	4.3 nm	Microbial viability assessment	Lin et al. (2018)
Beer yeast powder	Hydrothermal	Less than 5 nm	Imaging of bacteria	Gao et al. (2019)
Citric acid and urea	Microwave	2.4 nm	Antioxidation	Ji et al. (2020)
Citric acid and ethylenimine	Microwave	2.4 nm	Detection of Fe(III) Ions	Arvapalli et al. (2020b)
Chitosan and different unsaturated amides and carboxylic acid	Hydrothermal	2.6 ± 0.9 nm	Bioimaging, antioxidant	Wang et al. (2017)
Phenylenediamine	Hydrothermal	4.92 nm	Antioxidant	Gao J. et al. (2020)
Citric acid and urea	Microwave synthesizer	2.4 ± 0.3 nm	Radicals scavenging	Zhang et al. (2017)
Carbon fiber powder HNO3	Hydrothermal	1.8 ± 0.3 nm	Bioimaging	Liu et al. (2019)

As precursors for arc discharge, laser ablation, and high-energy ion beam radiation, carbon-based cement and graphite powder have been extensively used (Nguyen et al., 2015). Arc discharge, high-energy ion beam radiation, and laser ablation have all made extensive use of precursors such graphite powder and carbon-based cement (Li et al., 2012). For top-down CND synthesis, several approaches have been investigated. By oxidizing carbon nanostructure aggregates with a strong acid, chemical oxidation introduces hydrophilic functional groups containing oxygen. For top-down CND synthesis, several approaches have been investigated. Chemical oxidation is a technique that introduces hydrophilic functional groups containing oxygen into carbon nanostructure aggregates by oxidizing them with a strong acid. The re-entry of the carbon nanostructures into the fluid is facilitated by their increased solubility in water. The oxidized carbon nanostructures were made soluble in water by Xu et al.'s extraction of soot from arc-discharged materials, which allowed for their release into the solution. When soot was extracted from arc-discharged materials, 3.3M HNO_3_ was used to oxidize Luminescent CNDs and the black slurry was purified by gel electrophoresis after extraction with NaOH solution. Redox potentials strong enough to oxidize C-C bonds or water to produce oxygen radicals were used in electrochemical procedures. Ding et al. developed CNDs by using multi-walled CNTs generated by chemical vapor deposition as electrodes. This method’s practical and financial benefits prompted several developments regarding the composition of electrodes and carbon sources. Blue-fluorescent CNDs were created by cycling multi-walled CNTs on carbon paper in tetrabutylammonium perchlorate between −2.0 and +2.0 V. Higher CNDs emerged from lower current levels, and their size depended on the current density between 20 and 180 mA cm^2^ (Roy et al., 2015).

Additionally, investigated are solvothermal and hydrothermal cutting techniques. Among the first to use these techniques were Pan et al (2010), who used graphene sheets and hydrothermal cutting at 200 °C for 10 h to create CNDs. By using leeks in a one-step pyrolysis and hydrothermal treatment process, blue and green, fluorescent CNDs were created. This allowed for easy reaction condition modification and the production of tunable PL (Shi et al., 2016). Intercalation techniques are another common top-down strategy. These techniques usually entail the incorporation of an element or compound into carbon sources, e.g., graphite sheets. Lin et al. used potassium intercalation to create hydrophilic CNDs from CNTs and graphite flakes. When the CNT walls encountered ethanol, the combination of intercalated K atoms and graphene sheets broke down the barriers. The walls were further broken down to CNDs, among other byproducts, produced by ultrasonication. High degrees of accuracy are expressed by other top-down techniques. Graphene was sliced into desired sizes by Ponomarenko et al. using ultrahigh-resolution beam lithography. Li et al. produced CNDs directly by ultrasonic treatment of activated carbon mediated by hydrogen peroxide. Liu et al. vortexed commercial graphic nanoparticles in ethanol and water, then centrifuged the mixture at 2000 RPM for 30 min to provide a supernatant of sp^2^ CNDs with an ideal crystalline structure (Khan et al., 2021).

There have also been reports of the synthesis of hydrophilic, hydrophobic, and even amphiphilic carbon dots via microwave-assisted synthesis (De Medeiros et al., 2019). Mitra et al. (2012) described a relatively straightforward one-step microwave-assisted synthesis of hydrophobic CDs from the Pluronic F-68 (PF-68) block copolymer, which is composed of polyoxyethylene, polypropylene, and polyoxyethylene (PEO–PPO–PEO). Jaiswal et al. (2012) created CDs by caramelizing PEG under microwave assistance; in this case, glycol served as the CDs passivating agent and carbon source. Similarly, Zhai et al. (2012) reported pyrolytic reactions between amines and citric acid aided by microwaves. Ma et al. (2012) reported the use of ammonium hydroxide and glucose as a precursor in the ultrasonic synthesis of N-doped CDs. Dang et al. (2016) reported CDs on a massive scale by applying ultrasonic treatment to an oligomer polyamide resin, which served as the carbon source (Sivasankarapillai et al., 2020).

CDs are also extensively produced with the help of pulsed laser ablation method. To create CNDs with UV emission from a graphite target, a PLA (Pulsed Laser Ablation) technique is devised. The CNDs’ graphitic carbon cores are disclosed by TEM, XRD, Raman and XPS data, while their surface groups are functionalized with basic oxygen-related groups. As a result, there is very little excited electron trapping by surfaces and imperfections. Consequently, the CNDs display three sub-bands of intrinsic UV emission at 305 nm, 325 nm, and 335 nm. This study presented a novel method for creating U: V-emissive CNDs, which will greatly advance the use of CNDs in optoelectronics (Yang M. J. et al., 2021).

Direct pyrolysis, often referred to as the pyrolytic process, is a method frequently used in the synthesis of CDs. It entails carbonizing carbon precursors at high temperatures. To degrade the precursors at the nanoscale, a powerful acid or alkali is used. This pyrolytic process can use leftover fruit peels, any kind of juice, carbon-based chemicals, or anything that contains carbon as its carbonaceous source. Zhou et al. illustrated producing CDs using fresh watermelon peel as the starting material. Filtration and centrifugation were used to purify the final product after the peel was carbonized for 2 hours at 220 ^o^C. The particles had significant blue luminescence and were stable and soluble in water (Zhou et al., 2012). High quantum yield and good water solubility CDs are synthesized at 200 ^o^C using citric acid and glutathione as starting ingredients, as presented by Zhuo et al., (2015) and these particles had emission behavior that was independent of excitation and produced blue fluorescence. The addition of ligands and continuous stirring of the fluid, and the introduction of chemical bonds with amidation reactions or electrostatic interactions, are the factors responsible for the modification in the behavior of CDs.

Modification of the nanostructure and electrical distribution of CDs can be accomplished by doping them with heteroatoms, fluorescence may be regulated by nitrogen doping, and excitation-independent behavior can be exploited to infer passivated surface states (Arvapalli et al., 2020b; Gao J. et al., 2020; Wang et al., 2017; Zhang et al., 2017). Furthermore, N-doped carbon dots have been synthesized utilizing bee pollen as the carbon source by the use of hydrothermal carbonization techniques. It was found that the utilized method produced a good yield of 3 g of CDs from 10 g environmentally friendly precursor. Wang F. et al. (2010) employed a straightforward, one-step process to create CDs. The procedure involved loading 1.5 g of 1-hexadecylamine and 15 mL of octadecene into a three-neck flask, heating it to 300 ^o^C with an argon flow. After adding 1 g of CA to the mixture and stirring constantly for 3 hours at 300 ^o^C, the mixture was purified by precipitation and three acetone washes. The final product developed was CDs that were extremely soluble in ordinary non-polar organic solvents (Sivasankarapillai et al., 2020).

### 2.2 Bottom-up synthesis

To create CNDs from biological material, bottom-up approaches have been completely established as an alternative economic structure (Table 1). CNDs below 10 nm that are photoluminescent and persistent in an aqueous solution are often the product of the original components and circumstances. Gianneli et al. heated organic ammonium and citric acid (CA) monohydrate hydrothermally to produce 7 nm CNDs after acetone washing and filtering. Urea, ethanolamine, cysteine, glycine, ethylenediamine, and other organic materials have all been used in the synthesis of hydrophilic CNDs since the first CNDs were reported to have been produced. Zhu et al. synthesized PEG-200 and a dissolved saccharide using microwave pyrolysis for the first time. The size of the CNDs increased with reaction time when this solution was cooked in a 500 W microwave oven. Microwave pyrolysis increases the yield of CNDs, decreases side reactions, and shortens process times (Cayuela et al., 2016). Nevertheless, certain CNDs made with this technique exhibit reasonable luminescence and blue emission (Das et al., 2014). Qu et al. heated inorganic ions and carbohydrates for a few minutes to produce CNDs. Comparable techniques include heating organic molecules in solution for a duration ranging from seconds to minutes at 300–900 W. Reactants include PEG-200, polysaccharides containing inorganic ions (such 4,7,10-trioxa-1,13-tridecanediamine, or TTDDA), citric acid and amino acids.

For a microwave heating procedure, Stefanakis et al. used aqueous solutions of urea and either 6-aminocaproic acid or citric acid. CNDs were produced using microwave-cooking solutions. It was predicted that citrate molecules would form CNDs. The surface groups were made up of additional organic molecules, resulting in a hydrophilic CND. To create pure CNDs, the product is often dialyzed, centrifuged, or ultra-filtered once it is acquired. Polymers might be added by surface passivation, which usually takes minutes. Following the microwave synthesis of lipoic acid, citric acid, and urea, Chen et al. generated N, S-co-doped CNDs with OH- and NH surface groups, which made them hydrophilic (Chen et al., 2013). As determined by XPS, CNDs with strong graphite components and sp^2^ character are produced by microwave pyrolysis and laser ablation. The fact is that precursors are heated and carbonized in an inert environment during the process of direct thermal decomposition, which has produced a range of CNDs. Gianneli et al. developed CNDs in 2008 utilizing a direct thermal breakdown method; they had an asymmetric shape and were only stable in organic solvents like DMSO and DMF. After using a different method of thermal breakdown, which included combining citric acid with sodium 11-amminoundecanoate, heating the combination, and centrifuging the mixture, hydrophilic CNDs were produced. The end result was the production of core-shell structured CNDs with an overall size of 10–20 nm and a core size of 5–10 nm. Carbon and nitrogen sources were provided by citric acid and tris(hydroxymethyl)aminomethane to produce nitrogen-doped CNDs (N-CNDs) in a single process (Xu et al., 2015). The template synthesis method was utilized to create CNDs from monodisperse spherical mesoporous silica particles. After thermal decompression and template removal, organosilane was deposited into the pores to produce spherical CNDs (He et al., 2020).

**FIGURE 3:**
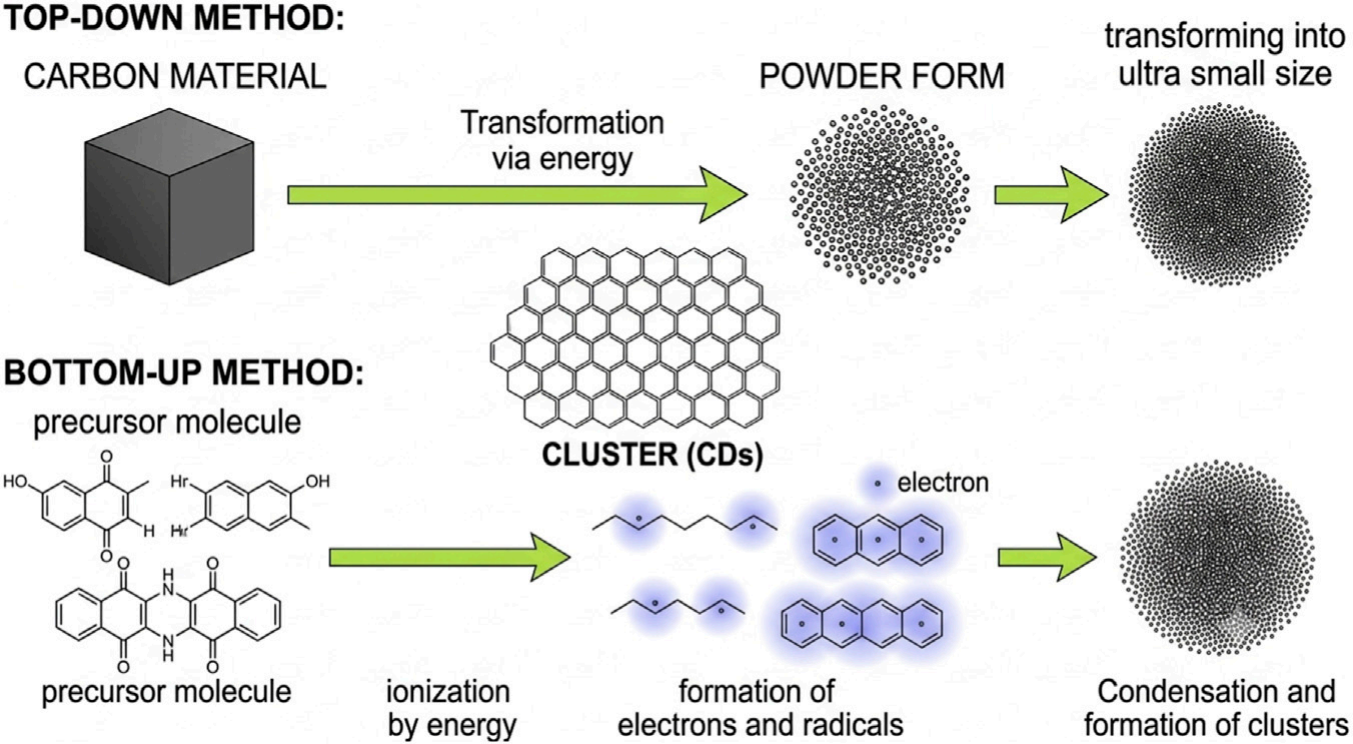
Bottom-up and top-down methods for synthesizing CNDs (Sharma and Das, 2019).

Liu et al.'s assisted synthetic technique uses formaldehyde or phenol resins as carbon precursors and polymer/silica composites as carriers. Heating facilitated the formation of carbon/silica blends, which, by the etching with NaOH, yielded CNDs. The oxidation, neutralization and purifying processes that came next were difficult and time-consuming. Li et al. used nanoreactors composed of silica spheres with holes ranging in size from 2 nm to 50 nm, which simplified this operation. They are stable, PL and comparatively nontoxic CNDs that do not require any further purifying processes. Because concentrated H_2_SO_4_ is a potent dehydrator, it has also been used to create a variety of CNDs. This procedure, which includes adding sulfuric acid to sucrose solution, neutralization, dialysis, and neutralization, was initially published by Zhang et al. The molecular weight of pure CNDs was used to distinguish them based on their photoluminescence behavior 2 nm CNDs emitted green light, whereas 5 nm CNDs were photoluminescent following PEG2000 N passivation. Other dehydration syntheses may use organics such as bovine serum albumin, ethylene glycol, or ethylenediaminetetraacetic acid (EDTA). Some employ heat as well; reaction temperatures can range from 40 ^o^C to 200 ^o^C. A plethora of other techniques have been devised to produce CNDs with a consistent size distribution and comparatively elevated purity. Kim et al. created an atomic carbon beam using ethylene gas and Ar plasma, and they carved graphene into CNDs using thermal plasma jets. After dissolving commercial coffee in 90 ^o^C water and centrifuging it at 14,000 RPM, Jiang et al. were able to extract approximately 4.4 nm CNDs by gel filtration chromatography. Li et al. employed ultrasonic treatment to synthesize as shown in Figure 3 (Shi et al., 2016).

**FIGURE 4:**
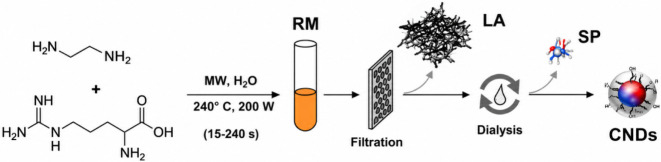
Diagram for the workup and synthesis process helped by microwaves (MW) (Rigodanza et al., 2021).

Usually, four sequential phases in the development of CNDs for optoelectronic application involve (i) tiny organic molecules aggregating; (ii) forming a dense core with an extended shell; (iii) the shell collapsing; and (iv) the core aromatizing (Table 2). Additional heating causes the core to aromatize but does not affect the emissive behavior. They introduced five new CNDs, all of which have an amine-rich ligand shell covering an electron-dense core; as a result, post-synthesis CND functionalization is not necessary to produce a core-shell structure (Figure 4) (Rigodanza et al., 2021).

**TABLE 2:** Comparison of carbon dot synthesis methods and their optical/optoelectronic characteristics.

Synthesis method	Typical size (nm)	Optical characteristics	Advantages	Limitations	Suitable optoelectronic applications
Hydrothermal	2–10	High photoluminescence, tunable emission, good quantum yield	Simple, eco-friendly, inexpensive	Long reaction time	LEDs, bioimaging, sensors, solar cells
Microwave-assisted	2–8	Bright fluorescence, high quantum yield, excitation-dependent emission	Very rapid synthesis, energy efficient	Limited scale-up	LEDs, fluorescent probes, sensors
Laser Ablation	2–6	High purity, stable emission, narrow size distribution	Chemical-free synthesis	Expensive equipment, low productivity	High-performance optoelectronics
Electrochemical	2–8	Tunable emission through voltage control, excellent surface functionality	Mild synthesis conditions	Low production yield	Electrochemical sensors, photovoltaics
Direct Carbonization	3–10	Good photostability, precursor-dependent fluorescence	Low cost, green synthesis	Poor size uniformity	LEDs, photocatalysis, solar cells

Along with preparation, the effect of applied voltage on CD size has been studied. Numerous papers describe the electrochemical method of producing CDs, such as Bao et al. (2011), who detailed the fluorescence behavior and electrochemical tuning of CDs. CDs have been successfully created from low-molecular-weight alcohols (Deng et al., 2014). In a basic medium, alcohols were transformed into carbon particles using the electrochemical carbonization process. Hou et al. (2015) reported an electrochemical process that produced CDs in a single pot and was utilized to detect mercury ions. Zeng et al. (2019) developed superior CDs that, by using a unique bio-electrochemical process, were able to enhance their electrical output and demonstrated outstanding catalytic performance (Sivasankarapillai et al., 2020). The viability of studying microwave-assisted hydrothermal techniques for synthesis has attracted attention. Wang Q. et al. (2011) used graphene oxide as the precursor in a hydrothermal process aided by microwaves to create CDs with favorable luminescence characteristics. Lately, Zheng et al. (2017) presented an additional quick technique that suppresses the quenching of the produced CDs fluorescence by employing N-(β-aminoethyl)-γ-aminopropyl trimethoxysilane.

Biomolecules may also be employed in synthesis. To create luminous CDs in the presence of monopotassium phosphate, Yang et al. (2011) employed glucose. The monopotassium phosphate in this system functioned as a regulating component to adjust the emission spectra. Additionally, biomolecules are employed as synthesis’s precursors. To create luminous CDs in the presence of monopotassium phosphate, (Arvapalli et al. 2020a) employed glucose. The monopotassium phosphate in this system functioned as a regulating component to adjust the emission spectra. Similarly, Chen et al. (2016) described a successful hydrothermal process that synthesized lignin. Zhang et al. (2018) created chiral CDs using a hydrothermal process in one step. The scientists discovered that the CDs might affect the physiology of mung bean plants by using citric acid and cysteine (cis-form) as carbon sources (Sivasankarapillai et al., 2020).

## 3 Optical properties of CDs

**FIGURE 5:**
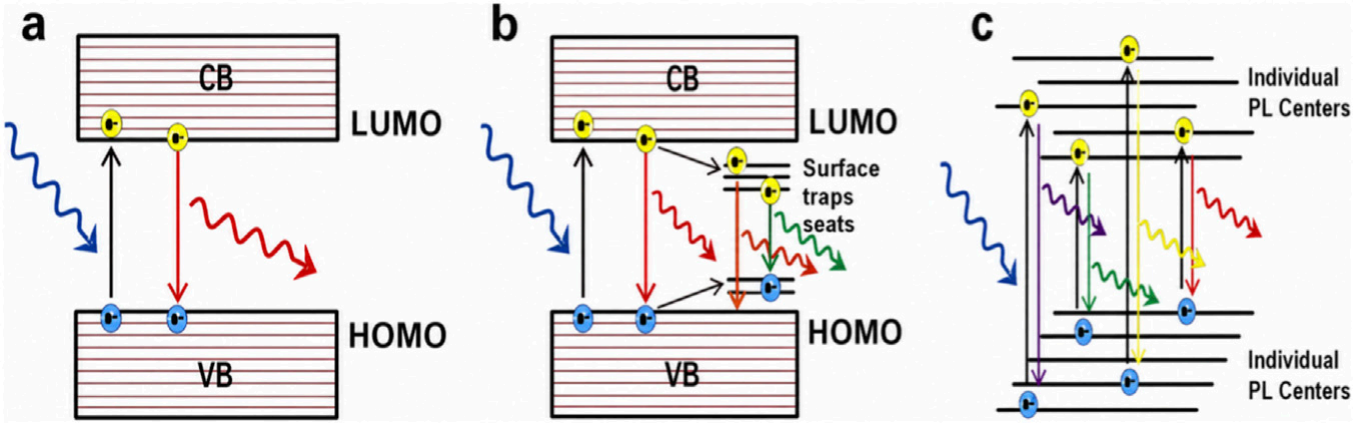
Scheme of the various photoluminescence mechanisms **(a,b)** and their unique characteristics **(c)** (Behi et al., 2022).

CNDs have drawn a lot of interest in the last decade. From a fundamentally applicable perspective, PL has attracted the interest of several researchers and is considered one of the most promising behaviors of CNDs (Kwee et al., 2021). PL refers to fluorescence, not formed by heating (Greenham et al., 1995). CNDs with distinct ratios of nitrogenous or oxygen-containing groups can arise from nucleation and development under various reaction circumstances and these CNDs have their distinctive PL properties (Figure 5). The excitation wavelength, quantum yield, and PL of CNDs are determined by their synthetic conditions, which include temperature, reaction time, and precursor materials (Bayan and Karak, 2019; Liu et al., 2015; Shen and Liu, 2016; Wang et al., 2016; Xu et al., 2015). The CNDs’ average UV absorbance is in the range of 330–420 nm. It has a tail in the visible area and is affected by photon harvesting. However, a 250–300 nm band may be present in CNDs due to * bond and ketone group transfer. Their emission wavelength typically falls between 400 and 600 nm, depending on their size (Li et al., 2012). Sun et al. have proven that the PL intensity of CNDs is related to their size. CNDs of different sizes and synthesis methods have been seen in the UV to NIR range. The transition gap between the highest occupied molecular orbital (HOMO) and lowest unoccupied molecular orbital (LUMO), which is inversely proportional to size, contributes to the size dependency of CNDs (Roy et al., 2015). Using gel electrophoresis and HPLC, CNDs with uneven size distributions were able to measure their peak lightness PL of 1.2 nm in the UV and 1.5–3.8 nm in the visible and near-infrared ranges (Roy et al., 2015).

Furthermore, the PL of 30–50 nm CNDs was lower than that of 10 nm CNDs, which can be attributed to the smaller CND’s greater PL due to their larger surface area to volume ratio. After hydrothermally cutting graphene sheets, CNDs with a size distribution of 5–17 nm and a matching PL wavelength of 450–486 nm were produced. The transformation of the CND from circular to polygonal shape, which raised the PL energy, was the cause of this reversal of the quantum size effect (Roy et al., 2015). Therefore, even across various sizes and shapes, the edge type of differently manufactured CNDs can affect PL. When λex was 470 nm, CNDs made from flower petals emitted a bright green light at 510 nm and showed absorptions from 220 to 400 nm. This might be because the band gap is being lowered by emissive flaws and a high graphite content (Paquin et al., 2015). The emission spectra of coffee-ground-synthesized CNDs changed from 400 to 600 nm, indicating their λex dependency (Li et al., 2012). The surface and core functional groups, as well as surface electronic state changes, can cause CNDs to become excited (Zeng et al., 2017). This characteristic sets them apart from other nanomaterials such as nanoclusters, Au-nanodots, and SQDs (Roy et al., 2015). Li et al. used hydrazine hydrate to decrease graphene oxide and then functionalized the surface with PEG.

The modified CND released photons at a lower wavelength (390–468 nm) when it absorbed several longer wavelength photons (600–800 nm) simultaneously or sequentially. Because CNDs absorb light poorly, oil-synthesized CNDs and CA-CNDs exhibited adjustable emissions that reduced fluorescence intensity with increased excitation (Table 3). This suggested that long λex may be employed to be less hazardous *in vivo*, limit background noise, and reach deep tissues CNDs produced from petals exhibited a red shift, an increase in PL intensity, and a subsequent drop in λex from 430 to 490 nm. Additionally, the average fluorescence lifetime of CNDs was 5.8934 ns (Paquin et al., 2015). Excited from 250 to 600 nm, hydrophobic N-doped CNDs showed a red-shifted emission that peaked at λex of 390 nm (Cayuela et al., 2016). They also demonstrated up conversion, a feature of CNDs that enables lower energy excitation of live cells, hence reducing harmful effects and aiding in interference elimination. When the excitation wavelength was altered from 700 to 900 nm, the N-doped CNDs exhibited a red shift and a narrowing of their emission peak at 500 nm (Khan et al., 2021).

**TABLE 3:** Different types of CDs are used in luminescence depending on the mode of excitation.

Types	Mode of excitation	Importance/applications	Presence of CDs	Ref.
Radioluminescence	Ionizing radiation	Scintillators, biomedicine, dosimetry	CQDs	Stellmer et al. (2015)
Photoluminescence	Photon	LEDs, display panel	CQDs	Gupta et al. (2021)
Triboluminescence	Frictional and electrical forces, mechanical energy	Sensor, aerospace, defense sector, civil construction	CNDs	Olawale et al. (2011)
Chemiluminescence	Chemical process	Pharmaceutical, clinical, analytical, gas analysis	GQDs	Iranifam (2014)
Cathodoluminescence	Cathode ray/electron beam	Pharmaceutical, mineralogy, display	SQDs	Barbin (2013)
Bioluminescence	Biological process	Imaging, dairy industries, biocidal disinfection	CQDs	Brock (2012)

The charge-transfer chemistry of CNDs, which allows them to function as either electron donors or acceptors, is one of their most remarkable characteristics. Several supramolecular structures with varying dimensions have been created by fusing CNDs with mesoporous surfaces or molecular and particulate building pieces. Charge transfer to or from photoexcited CNDs is how they function. Generally speaking, CNDs have developed into adaptable building blocks for supramolecular structures with specific electrical characteristics, indicating their potential application in low-cost charge-transfer active materials for technological devices (Cadranel et al., 2019). The optical and antioxidation characteristics of CNDs are studied by doping them with phosphorus (P) and boron (B) in varying amounts. Following doping, high P%-CNDs’ UV–vis absorption exhibited a little blue shift (348-345), whereas high B%-CNDs’ absorption revealed a modest redshift (348–351 nm). A study on the scavenging of 2,2-diphenyl-1-picrylhydrazyl (DPPH) radicals was carried out for every CND. The highest scavenging ability was found to be indicated by the high B%-CNDs. This work may provide guidance in the development of CND structures in the core and surface functional groups, as a helpful method for modifying the optoelectronic properties of CNDs for photoluminescence and antioxidation applications (Azami et al., 2023).

Cl-CNDs, or chlorine-doped carbon nitride dots, are also claimed to be produced by thermally treating guanidine hydrochloride, making them both luminous and phosphorescent. The phosphorescence quantum yield of the Cl-CNDs is 2.32% and their phosphorescence lifetime is 657 ms when they are triggered at 360 nm in ambient conditions. Since Cl-CNDs are inexpensive to produce and have changeable optical properties, they may find application in security encryption and optoelectronic systems (Patir and Gogoi, 2022). The green fluorescence nature of CNDs was evaluated at 510 nm for maximum emission using photoluminescence and ultraviolet-visible absorption spectra. In the current study, the scientists employed an easy-to-use and low-cost technique to synthesize vivid green PL CNDs from soft coconut water using acid-assisted ultrasonic with an excitation wavelength of 450 nm. The fluorescence quantum yield of the carbon nanotubes (CNDs) having a carbon core and chemical groups all over their surface was 60.18%. These results and observations offer a foundation for a range of upcoming investigations into the use of CNDs in cell imaging and optoelectronics (Manoharan et al., 2020).

It was described as an oxygen-free synthesis of CNDs with exceptionally low oxygen contents based on the pyrolysis-induced decarboxylation of aromatic carboxylic acids. The creation of CNDs nearly usually involves a certain kind of oxidation process. With an absolute quantum yield that may reach 80%, the CNDs show very strong excitation-independent PL in the visible spectrum’s yellow to orange region. This novel synthetic method, which is predicated on the non-oxidative pyrolysis of ACAs, sheds light on the synthesis of highly luminous CNDs and emphasizes the necessity of giving oxygen’s impact on the chemical, optical, and electrical aspects of CNDs more thought. Using the pre-made CNDs as a stand-alone color filter, vivid orange light was produced (Lee et al., 2021).

The study focused on polyethyleneimine CNDs (PEI CNDs) with concentration-dependent PL properties and unique excitation using a one-pot hydrothermal technique. The findings demonstrated that L-Glu and PEI might be directly connected to the CNDs, allowing the CNDs to handle rich amniotic groups. The carbon core of the PEI CNDs is primarily responsible for the excitation-independent blue PL behavior at low concentrations (0.1 mg/mL and 0.2 mg/mL). The UV–Vis absorption at 350 nm shows a considerable increase when the concentration of PEI CNDs rises from 1 mg/mL to 10.0 mg/mL, and the absorption peaks broaden as the concentration of PEI@CNDs increases. Green, cyan, blue, and greenish-yellow are displayed in the PL colors. The color coordinates of (0.30, 0.31) and (0.39, 0.40) could be produced by combining 10.0 mg/mL of PEI CNDs with polymer PVA and employing light-conversion films of PVA containing PEI CNDs to combine with the blue LEDs to create WLEDs with white and warm white emissions. Results demonstrated that the PEI CNDs may be employed in WLED fabrication, and they show tremendous promise in the field of optoelectronics (Zhang et al., 2023).

A variety of functional groups, including C-O, C=O, C-O-C, and C-N, are attached to the surface and edges of the carbon nucleus of N-CNDs. This was demonstrated by multi-color fluorescent N-CNDs that were created using a microwave technique with CA and L-glutamic acid as carbon sources. Deionized water was used as a dispersion agent. The generated N-CNDs can produce green (546 nm), orange (617 nm), and blue (445 nm) fluorescence, depending on the raw material ratios of 1:1,1:2 and 1:3. Variations in the N concentration doped into N-CNDs can result in distinct surface/edge states, and by adjusting the N concentration in CNDs, one can adjust the wavelength of the PL emission from N-CNDs. The number of C-O groups on the surface or edges of N-CNDs is correlated with their PL intensity. When the concentration of N in N-CNDs increases, the wavelength of the PL emission from these N-CNDs exhibits a redshift (Li et al., 2021).

## 4 Application of CDs in optoelectronic

**FIGURE 6:**
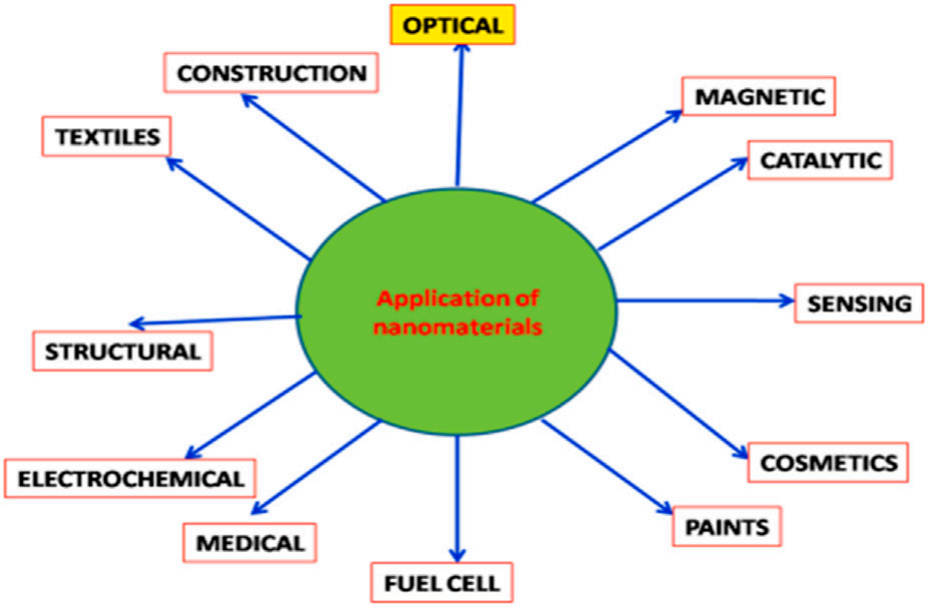
Different applications of carbon materials but focus on optical applications (Gupta et al., 2021).

CNDs have many benefits, such as abundant and readily accessible raw materials, low cost, multicolored photoluminescence, convenient synthesis, excellent biocompatibility and acceptable renewability. Researchers synthesize these new nanomaterials which are valuable and environment-friendly with novel applications (Figure 6). CDs also show various applications in optoelectronics (Kwee et al., 2021).

### 4.1 Carbon dots as a sensitizer in DSSC

The exceptional electron-donor and acceptor electrochemical properties of CDs (Kwon et al., 2014; Stellmer et al., 2015), in addition to producing both electrochemical and chemical luminescence (Li et al., 2015), give them a broad range of possibilities in sensors, optoelectronics, and catalysis. Numerous solar cells have made use of CDs’ exceptional light-harvesting and conducting qualities (Table 4). As CDs frequently exhibit excellent optical absorptivity throughout a broad visible light spectrum, they might be used as a substitute sensitizer in DSSCs. The most sophisticated DSSCs available today usually use sensitizers based on Ru. The problem is that while Ru-based sensitizers work well most of the time, they are difficult to synthesize and rely on the pricey and rare Ru-metal core, therefore there is an urgent need for a sustainable, eco-friendly alternative. Low current density may result from photo-charged surface recombination, which is also seen when CDs are employed as sensitizers (Gao N. et al., 2020).

**TABLE 4:** Sensitizer devices.

Classification	CND role	Device architecture	Optimized conditions	Explanation for performance	References
CQDs	Sensitizer	FTO/TiO2 NPs/CQDs/I3 –: I–/Pt	No optimization	Emissive sites acting as recombination centers, inferior charge injection properties	Mirtchev et al. (2012)
GQDs	Sensitizer	FTO/TiO2 NPs/GQDs/I3 –: I–/Pt	No optimization	Low affinity of GQD functional groups for TiO2	Yan et al. (2010)
CNDs	Sensitizer	FTO/TiO2 NPs/CNDs/I3 –: I–/Pt	No Optimization	IR light. Low performance due to high recombination rates with CNDs and poor electron transfer	Wang et al. (2012)
CQDs	Electron donor	No device made	No optimization	CQDs promoted photosynthesis by enhancing the electron transfer process; chloroplasts absorbance profile overlaps with CQDs emission profile (390 nm excitation) resulting in electron transfer from CQDs to chloroplasts	Chandra et al. (2014)
CQDs	Electron acceptor/dono r	FTO/TiO2 NPs/RhB/CQDs/I3 –:I–/Pt	No optimization	CQDs act as electron/energy transfer bridge between RhB and TiO2 mimicking processes involved in photosynthesis; CQDs enhanced absorbance of RhB and acted as one-way bridge, effectively separating the charge carriers and suppressing recombination	Ma et al. (2013)
GQDs	Electron acceptor	ITO/PEDOT:PSS/P3HT:GQDs/LiF/Al	1 wt% GQDs	Depends on GQD wt%; Appropriate band alignment, improved optical properties and morphology (e.g., nanoscale phase separation) of composite film	Gupta et al. (2011)

### 4.2 Light-emitting diodes (LEDs)

Lately, several study teams have endeavored to use CNDs as an active component in optoelectronic apparatuses, such as light-emitting diodes (LEDs). The Liu and Ma groups showed off the electroluminescence of CNDs made using the noncoordinating solvent approach for LEDs, although achieving an acceptable brightness required an extremely high operation voltage (about 9 V) (Barbin, 2013; Iranifam, 2014). Additionally, phosphor-based LEDs with CNDs made of photonic crystals were disclosed by Chen and colleagues (Guo et al., 2012). Three colors; bright blue, orange, and warm white are rendered satisfactorily; nevertheless, the resulting CNDs only exhibited a small amount of absorption in the deep-blue (long-UV, 360–400 nm) range. As a result, the most popular base LEDs, Gallium nitride (GaN) blue LEDs, are not very effective for this system. Furthermore, aggregation of solid-state CNDs often leads to considerable light quenching, which would considerably restrict the performance of CND-based LEDs (Essner and Baker, 2017; Li et al., 2015).

LEDs are considered the lighting technology of the future because of their high stability, affordability, eco-friendliness and mass manufacture. As a novel kind of carbon-based QDs, CQDs seem to be one of the most promising substitutes for costly rare-earth-based phosphors and hazardous metal-based semiconductor QDs to speed up the development of electroluminescent full-color displays and lighting. Phosphor-converted LEDs (pc-LEDs) and electroluminescent LEDs are the two main types of LEDs based on CQDs. The term “pc-LEDs” describes the optical pumping of CQD-based phosphors using blue/NUV chips as the primary light source. When electrons and holes are introduced into CQDs for electroluminescent LEDs, they undergo radiative recombination, which functions as an active emission layer. Among these, the creation of electroluminescent LEDs with high efficiency is crucial for next-generation displays (Shi et al., 2022).

### 4.3 Solar cells (SCs)

CQDs have potential uses in solar cells (SCs), including dye-sensitized SCs, organic SCs, and silicon-based SCs, because of their broad spectrum of light absorption (Chandra et al., 2014; Mirtchev et al., 2012; Wang et al., 2012; Yan et al., 2010). Currently, the photocurrent and power conversion efficiency (PCE) of corresponding perovskite solar cells (SCs) may be greatly increased (from 8.81% to 10.15%) by adding a sub-monolayer layer of CQDs between the perovskite and the mesoporous titanium dioxide layers. This work demonstrated that hot electrons from the photo-excited perovskite may be collected because of their sluggish cooling (Shi et al., 2018). Additionally, by adding CQDs to stabilize MAPbI3 by grain boundary passivation, the PCE increased 17.59%–18.81%) and perovskite SC stability enhanced (Guo et al., 2019). Despite the paucity of studies on CQDs in the field of SCs, CQDs remain a viable alternative to inorganic halide perovskites, which are unstable and poisonous (Shi et al., 2022). Another instance is the heterojunction of p-type PbS colloidal quantum dots (CQDs) with n-type wide-band-gap semiconductors, such as ZnO or TiO_2_. It is anticipated that confinement within these nanostructures would lead to marginal phenomena, such as hot carrier collection and multiple exciton formation, hence raising the theoretical limit of conversion efficiency. In the end, this method may potentially result in the construction of a tandem-type cell that absorbs light in several solar spectrum areas thanks to CQD films (Malgras et al., 2017).

### 4.4 Phosphor-converted LEDs

Two distinct peaks at 448 and 548 nm are produced by the LED using CQDs as phosphors when compared to the PL spectra of the CQD solution. This phenomenon might be explained by the solid-state CQDs integrated with the LED lens transferring energy through light reabsorption (Kwon et al., 2014; Li et al., 2015; Yan et al., 2010). To create pc-WLEDs, blue/NUV LED chips and CQD phosphors are often mixed (Guo et al., 2012; Kwon et al., 2013). However, because of direct π–π interactions, CQD phosphors are limited to self-quenching in the solid state, which is insufficient for real-world uses. In this instance, phosphors have been embedded into solid matrices (such as polymers, starch, and silica xerogel) conventionally to provide solid-state illumination of CQD-based composites. The realization of high-performance multicolor pc-WLEDs still confronts significant obstacles since there are fewer multicolor CQDs with high QYs in solid states and because solution-processability is a concern. Effective singlet-component CQDs in solid states have been studied in the last several years. To create warm white light emission, for example, R/G/B-SBF-CQRs with high QYs up to 30%–46% have been successfully applied to LEDs. This shows promise for acting as a replacement for conventional rare earth phosphors and realizing industrialization in the future (Gao N. et al., 2020; Wang et al., 2012).

## 5 Current bottlenecks and future perspectives

Although carbon dots have significant optoelectronic potential, a number of outstanding material issues need to be resolved before commercial translation can take place:

### 5.1 Low reproducibility of emission properties

Because polymerization, carbonization, and passivation occur simultaneously during bottom-up synthesis, even small changes in batch temperatures or heating rates can drastically change surface topologies (Kwon et al., 2014; Rigodanza et al., 2021). To guarantee consistent optical performance from batch to batch, standardized, automated synthesis processes must be developed. Furthermore, in parallel tracking systems, integrating physics-based rendering with neural network embeddings is essential to maintain multi-source illumination color constancy despite these material variances (Xue et al., 2025).

### 5.2 Scale-up limitations

High quantum efficiency production techniques, such microwave or hydrothermal synthesis, are usually limited to tiny batch sizes (He et al., 2020). Thermal and mass-transport gradients are frequently introduced when scaling up to commercial levels, which widens particle size distributions and deteriorates color purity. This optical variance presents similar challenges to low-light imaging frameworks, where multiconditional diffusion probabilistic models (such as MCLL-Diff) are deployed to dynamically adapt and enhance contrast under poor visibility conditions (Chen et al., 2025).

### 5.3 Stability under device operating conditions

CDs are subjected to extended exposure to heat, moisture, and strong UV fluxes when incorporated into high-power LEDs or solar cells. In these circumstances, thermal desorption or photo-oxidation of the surface functional groups that passivate fault states may occur, resulting in irreversible luminescence degradation and device failure (Essner and Baker, 2017). To monitor macro-structural shifts and thermal anomalies linked to this degradation, highly precise diagnostic tools are required, such as using self-mixing interferometry with intracavity frequency-doubling solid-state lasers to trace micro-vibration signatures (Feng et al., 2026).

To accelerate the practical deployment of these materials in complex optical systems, research must also bridge the gap between material synthesis and advanced system-level calibration. In next-generation optoelectronics and light-emitting systems, single-view neural illumination estimation frameworks are now utilized to dynamically edit and adjust complex environmental lighting conditions for high-fidelity light field displays (He et al., 2026). Concurrently, to safeguard long-term stability and catch defects early on a industrial scale, photovoltaic module inspection platforms are implementing advanced electromagnetic induction-assisted scanning photoluminescence imaging (Hong et al.,2026). Bridging these materials-level improvements with systematic, automated imaging diagnostics will be key to unlocking the full potential of next-generation carbon dot optoelectronics.

Although carbon dots have recently emerged as versatile nanomaterials for optoelectronic applications, many of the fundamental concepts governing their optical performance have evolved from extensive studies on conventional semiconductor materials. Wide-bandgap semiconductors have served as benchmark systems for understanding the relationship between crystal quality, defect density, and radiative recombination. Previous investigations have demonstrated that synthesis parameters, including deposition temperature and precursor chemistry, significantly influence crystallinity, carrier transport, and photoluminescence efficiency. Likewise, improvements in epitaxial growth strategies and optimized buffer layers have been shown to enhance optical emission by minimizing structural defects and impurity incorporation. These studies have provided valuable insight into the design principles required for developing highly efficient optoelectronic materials, even though the materials themselves differ substantially from carbon-based quantum dots (Dai et al., 2006; Dai et al., 2007; Dai et al., 2011).

In comparison with inorganic semiconductor films, carbon dots offer several practical advantages, including low toxicity, abundant precursor availability, excellent aqueous dispersibility, facile surface functionalization, and solution-processable synthesis. Nevertheless, both material classes share common objectives in optoelectronic engineering, namely, maximizing light absorption, improving charge-carrier dynamics, reducing non-radiative recombination, and achieving stable photoluminescence. The development of multifunctional ultraviolet optoelectronic devices based further illustrates how careful control of optical and electronic properties can enable integrated light-emitting and photodetecting functions. Such advances provide a useful conceptual framework for interpreting the rapidly expanding applications of carbon dots in light-emitting diodes, photodetectors, bioimaging, optical sensing, and photovoltaic technologies, where similar optimization strategies are increasingly employed through heteroatom doping, surface-state engineering, and hybrid nanocomposite design (Dai et al., 2009; Dai et al., 2010).

## 6 Conclusion

The main carbon dot synthesis techniques are critically compared in this review, which also shows how the synthesis pathway has a significant impact on the structural, optical, and optoelectronic characteristics of carbon dots. While laser ablation produces extremely pure CDs appropriate for cutting-edge optoelectronic devices despite its greater cost, hydrothermal and microwave-assisted techniques often offer superior photoluminescence performance and high quantum yields with comparatively easy processing. While direct carbonization is a cost-effective and ecologically benign method, improvements in size uniformity and repeatability are still needed. Electrochemical synthesis provides exceptional control over surface chemistry and electrical characteristics. In addition to highlighting the benefits and drawbacks of each synthesis technique, this review’s comparative analysis shows a direct correlation between synthesis conditions and device performance in LEDs, solar cells, sensors, and related optoelectronic applications. These discoveries offer helpful direction for choosing suitable synthesis techniques and point the way forward for the creation of high-performance carbon-dot-based optoelectronic materials.
